# Gastric lipomatosis treated by total gastrectomy: a case report

**DOI:** 10.1186/s40792-017-0404-1

**Published:** 2017-12-16

**Authors:** Shota Aoyama, Katsunori Ami, Akira Fukuda, Kenichiro Imai, Ja-Mun Chong, Masayuki Ando

**Affiliations:** 1 Department of Surgery, Tokyo Metropolitan Health and Medical Treatment Corporation, Toshima Hospital, 33-1, Sakae-cho, Itabashi-ku, Tokyo, 173-0015 Japan; 2 Department of Pathology, Tokyo Metropolitan Health and Medical Treatment Corporation, Toshima Hospital, Tokyo, Japan

**Keywords:** Lipomatosis, Gastrectomy, Ulcer, Gastric lipoma

## Abstract

**Background:**

Gastric lipomatosis is characterized by multiple gastric lipomas or a diffuse gastric infiltration of the submucosal or subserosal layer by the adipose tissue; diffuse-type gastric lipomatosis is an extremely rare condition. Here, we present the case of a patient with gastric lipomatosis treated by total gastrectomy.

**Case presentation:**

A 54-year-old man diagnosed with gastric submucosal tumor in 2008 was referred to our hospital for further examination and treatment in September 2016. Upper gastrointestinal endoscopy revealed a submucosal tumor with an associated ulcer on the anterior wall of the lower body of the stomach. A compressing mass was observed on the anterior wall of the greater curvature and the posterior wall of the stomach. Following a biopsy of the submucosal tumor and ulcer, lipoma without malignancy was diagnosed by microscopy. A giant gastric lipoma was suspected because endoscopic ultrasound revealed a high-echoic lesion on the antral wall that extended to the stomach. Therefore, total gastrectomy was performed, and gastric lipomatosis was confirmed by a histological examination of the resected specimen.

**Conclusions:**

Surgical treatment is a highly effective treatment for symptomatic gastric lipomatosis with extensive involvement or multiple lipomas and can be used for patient diagnosis.

## Background

Gastrointestinal (GI) lipomatosis is characterized by multiple, small, and asymptomatic lipomas, which are one of the most common submucosal tumors (SMTs) of the GI tract [1, 2]. Although GI lipomas are the second most common benign colonic tumors [1, 2], much less frequently described is the presence of multiple lipomas or intestinal lipomatosis; thus, they lack a clear diagnostic criteria because of few reports published in the literature. Moreover, gastric lipomas are rare, accounting for only 5% of all GI lipomas and < 1–3% of all gastric tumors [1–4]. Gastric lipomatosis is even rarer, with only eight cases previously reported [1]. Here, we present the case of a patient with suspected gastric lipoma associated with ulcer and bleeding who was treated by gastrectomy. A pathological examination of the resected specimen confirmed gastric lipomatosis. This is the first case of lipomatosis with ulcer and bleeding.

## Case presentation

Our patient was a 54-year-old man who was originally diagnosed with a gastric SMT in 2008. He underwent an annual follow-up by upper GI endoscopy at another hospital but was referred to our hospital for further examination and treatment in September 2016, during which his main clinical symptoms were epigastric soreness, general malaise, and melena. He had an unremarkable family history.

**Fig. 1:**
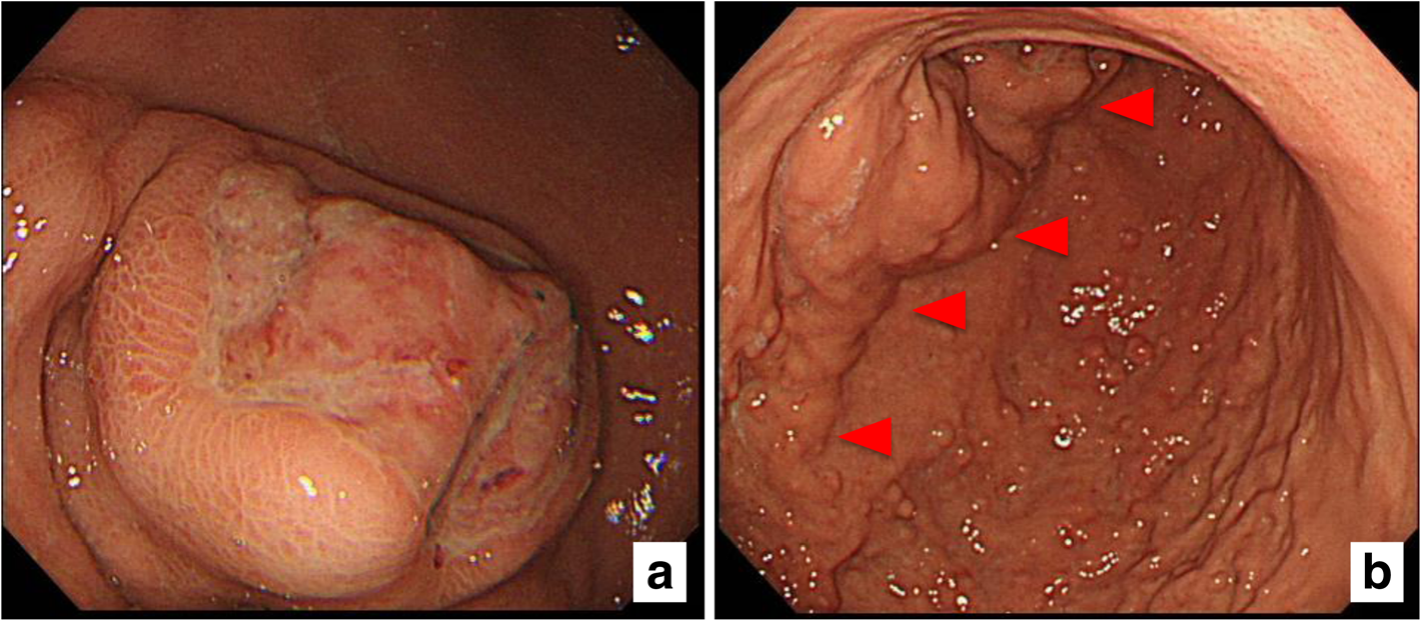
**a**, **b** Findings on upper GI endoscopy. An SMT and ulcer are present on the anterior wall of the lower body of the stomach (**a**). Multiple hyperplastic polyps are seen on the mucosa (**b**). There is no active bleeding from the SMT or ulcer. Red triangle indicates a part of the thickening of the stomach wall. Abbreviations: GI gastrointestinal, SMT submucosal tumor

On admission, he measured 161.6 cm tall and weighed 58 kg and his pulse was 115 beats/min, and blood pressure was 69/43 mmHg. There were no significant findings on abdominal examination, but laboratory analysis revealed a hemoglobin level of 5.6 g/dl. Other hematological and biochemical parameters were within normal limits. Chest X-ray, electrocardiography, and echocardiography results were normal. On admission, because he had advanced anemia and low blood pressure, he received blood transfusion (480 ml). On upper GI endoscopy, an SMT was identified with an associated ulcer on the anterior wall of the lower body of the stomach (Fig. 1a). There was an extrinsically compressing mass on the anterior wall of the greater curvature and the posterior wall of the stomach (Fig. 1b). However, no active bleeding from the SMT or ulcer was observed.

**Fig. 2:**
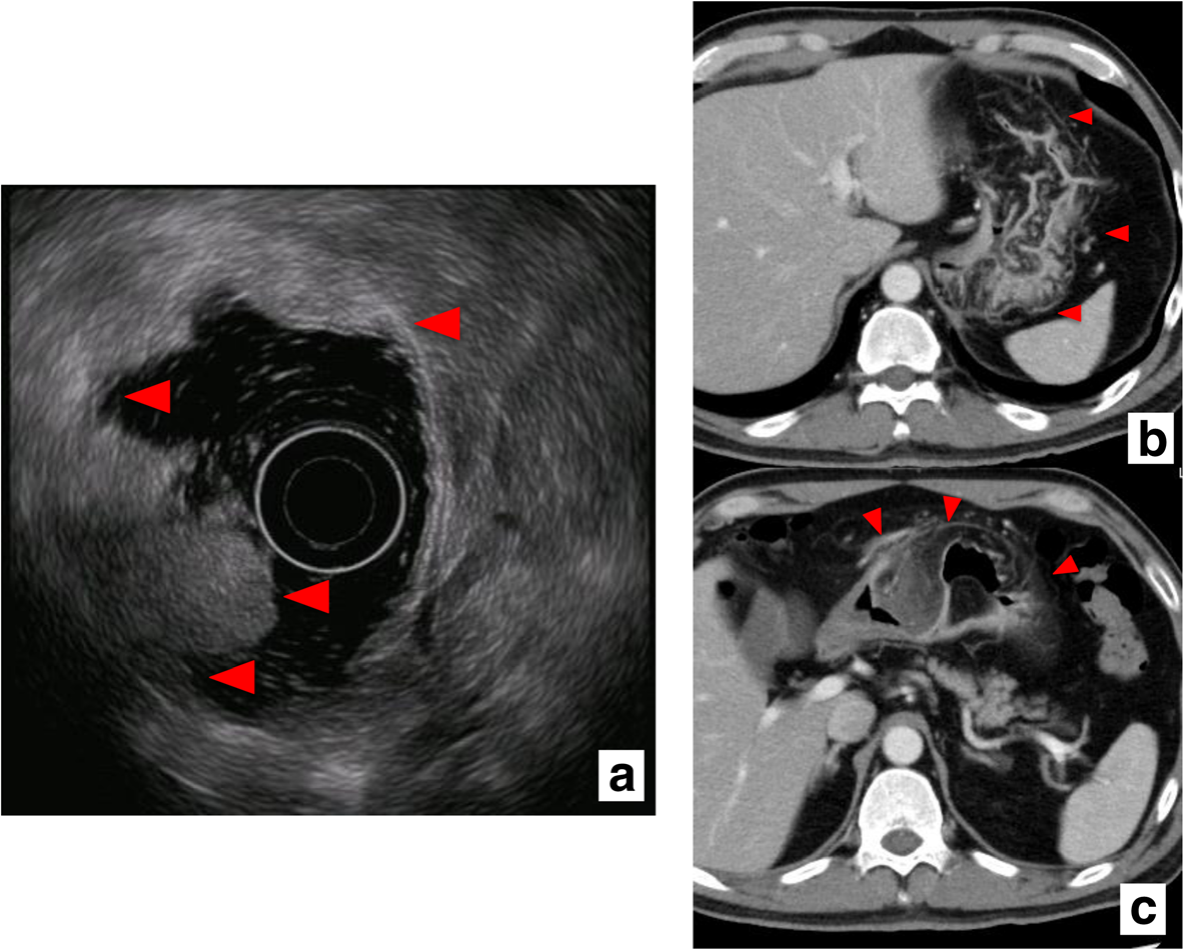
Findings on EUS and CT. EUS (**a**) and CT (**b**, **c**) findings are shown. EUS shows a high-echoic lesion in the antral wall submucosa (red triangle), extending to the stomach. CT shows a huge fat-containing mass lesion around the gastric wall, excluding the lesser curvature (red triangle). Abbreviations: CT computed tomography, EUS endoscopic ultrasound

**Fig. 3:**
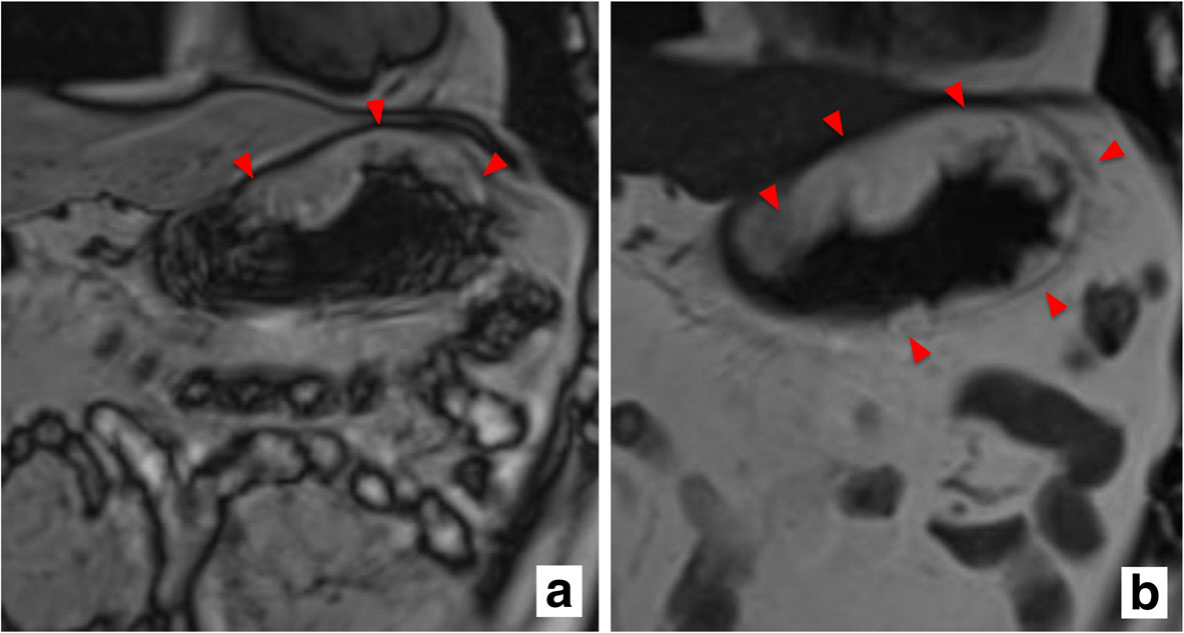
Findings on T1- and T2-weighted MRI. T1- (**a**) and T2-weighted (**b**) MRI findings are shown. There is a high-intensity lesion in the submucosa with a fat-containing mass on the entire wall of the gastric antrum and body, but excluding the lesser curvature (red triangle). Abbreviations: MRI magnetic resonance imaging

We diagnosed lipoma without malignancy based on the biopsy of the SMT and ulcer. Endoscopic ultrasound (EUS) confirmed a high-echoic submucosal lesion in the antral wall that extended to the stomach body (Fig. 2a), and computed tomography (CT) and magnetic resonance imaging confirmed a fat-containing mass spanning entire gastric walls of the stomach antrum and body, but excluding the lesser curvature, with a mass protruding on the anterior wall of the greater curvature (Figs. 2b, c and 3). CT images revealed no metastasis to lymph nodes or to other organs, and the serum tumor markers CEA and CA19-9 were within the normal limits.

**Fig. 4:**
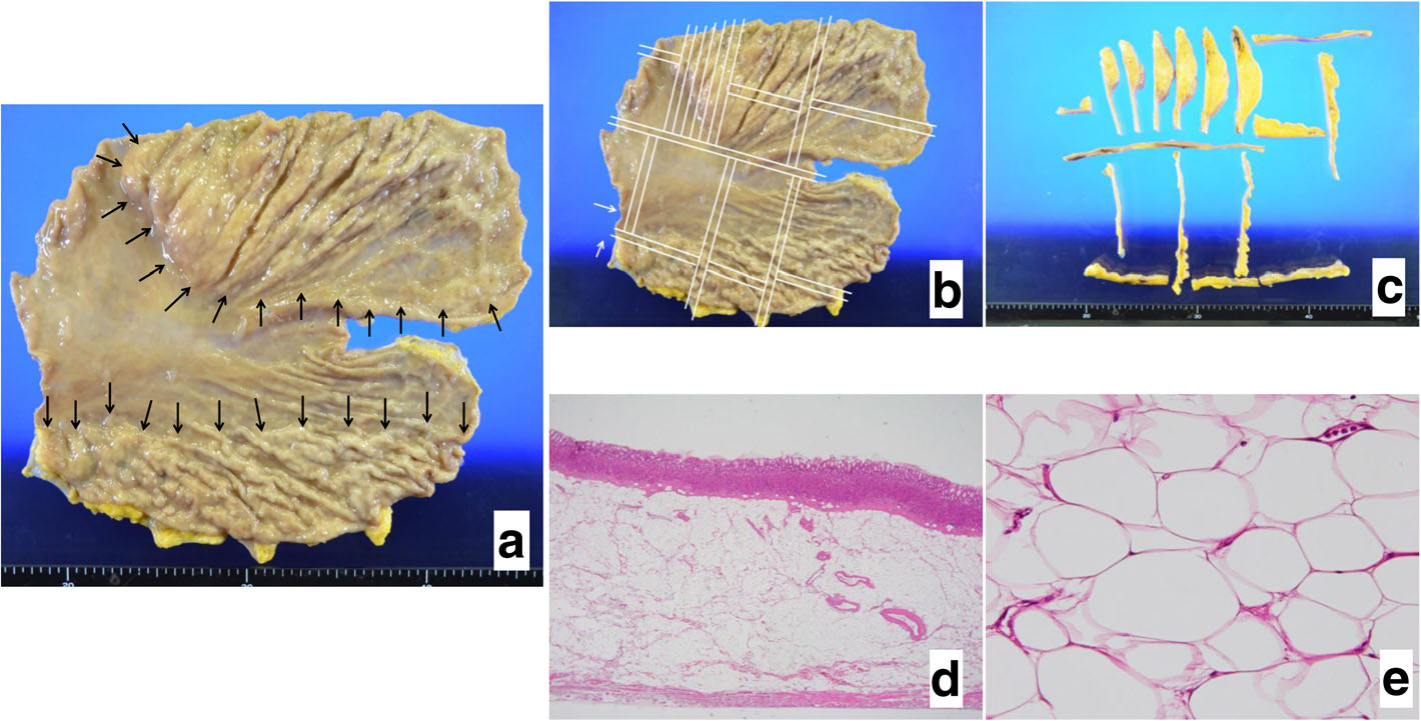
Pathological findings. The specimen is opened along the greater curvature (**a**). The mucosal surface is smooth and diffusely elevated by the submucosal mass, excluding the lesser curvature of the wall. We cut the specimen with white line (**b**). Gross pathology shows a yellowish adipose tissue (**c**), and microscopy shows adipocyte proliferation without nuclear atypia (**d**, **e**) or malignancy

Based on our findings, we suspected a giant gastric lipoma and proceeded to perform a standard total gastrectomy. We considered the execution of resection of the stomach, but we decided to perform total gastrectomy because the range of the tumor was unclear and the risk of recurrence could not be determined. The specimen was opened along the greater curvature, revealing a mucosal surface that was smooth and diffusely elevated by the submucosal mass, but with no involvement of the lesser curvature (Fig. 4). There was a compressing lesion associated with an ulcer on the anterior wall of the lower body. The gross pathology was of a yellowish adipose tissue with no fibrous capsules.

Gastric lipomatosis was confirmed by a histological examination of the resected specimen. Microscopy revealed adipocyte proliferation without nuclear atypia and mature adipocytes replacing the submucosal and muscle layers of the stomach. Lipomatosis was present only in the fundic gland zone, and there was no intestinal metaplasia or atrophic gastritis. Multiple hyperplastic polyps were observed on the mucosa overlying the area of diffuse lipomatosis. Immunostaining was negative for MDM2, CDK4, and p16. Moreover, there was no evidence of malignancy in the fatty lesion.

The patient recovered well following surgery and was discharged on the postoperative day 14. At the latest follow-up, he was continuing to do well and showed no evidence of recurrence in other organs.

### Discussion

Gastric lipomas are characterized by smooth, sharply marginated, and oval or spherical submucosal masses comprising well-differentiated adipose tissues surrounded by a fibrous capsule [1–4]. In contrast, gastric lipomatosis is characterized by multiple gastric lipomas or diffuse infiltration of mature adipose tissues into the gastric submucosal or subserosal layers. GI lipomas are extremely common benign colonic tumors, whereas gastric lipomatosis is particularly rare [2, 5–12].

Approximately, 90–95% of lipomas are submucosal, and the rest are subserosal [12]. Most gastric lipomas are small (4–9 cm) and asymptomatic, occur on the posterior wall of the antrum, and are incidentally detected on radiographic or endoscopic examination of the upper GI tract. Smaller lesions are rarely symptomatic, but large tumors can present with symptoms of gastric ulcer, including epigastric pain, nausea, vomiting, and upper GI tract bleeding. Indeed, the most frequent clinical manifestation is GI bleeding, which is due to the ulceration of the overlying mucosa in 50% of the patients [2, 12]. When a large lipoma is present, venous stasis is probably the single most important factor underlying mucosal ulceration, which may lead to acute, and sometimes severe, upper GI hemorrhage. Several authors have reported anemia as the primary indicator of large gastric lipomas [5–12]. In the present case, we suspect that a part of the tumor rapidly increased in size and led to the collapse of the mucosal surface with subsequent ulceration and bleeding.

CT is an excellent diagnostic tool because it allows the diagnosis of lipoma based on tumor fat density, precluding the need for an endoscopic biopsy. Indeed, a homogeneous mass with a fat density ranging between − 70 and − 120 Hounsfield units is considered pathognomonic of gastric lipoma [13]. Histologically, GI tract lipomas typically have well-differentiated adipose tissue structures. If a large submucosal tumor is detected on an endoscopic or upper GI examination, then a CT scan can be used to confirm the diagnosis and to inform therapy decisions. However, EUS is the most useful diagnostic tool for assessing neoplasia originating from the submucosa. In the present case, EUS showed a submucosal hyperechoic and homogeneous mass and diagnosed lipoma.

Although conservative treatment is preferred for asymptomatic solitary lipomas, surgical intervention should be considered for symptomatic lipomas associated with ulcers or non-fatty elements. Endoscopic polypectomy is an option for submucosal lesions that are smaller than 3 cm, but larger broad-based tumors have a higher risk of perforation using this approach. In our case, we decided to treat the patient by total gastrectomy for three main reasons: first, a large and symptomatic lipoma was present at surgery; second, although biopsy suggested lipoma, we could not completely exclude malignancy; and third, anastomotic leakage and tumor recurrence were possible if partial resection was performed.

Eight reports of gastric lipomatosis exist in the literature, of which five describe a detailed pathology (Table 1) [2, 5, 6, 8–12]. According to these reports, lipomatosis in the upper stomach can occur with multiple organ involvement or with multiple types of lipomatosis. However, no tumor has been reported in any other organ when lipomatosis occurs in the lower stomach. Most previous cases have reported multiple lipomas, and this is only the second case of gastric lipomatosis presenting as a diffuse lipoma [2]. In our case and in that by Jeong et al. [2], the cases not only had similar specimens and histological findings but were also diagnosed as diffuse-type lipomatosis. In our case, it was noteworthy that the lipomatosis was present only in the fundic gland zone, without intestinal metaplasia or atrophic gastritis. Furthermore, multiple hyperplastic polyps were observed on the mucosa overlying the diffuse lipomatosis. We hypothesize that a relationship exists between hyperplastic polyposis and lipomatosis in some cases.

**Table 1:** Case of gastric lipomatosis

Reference	Age	Sex	Site	Size	Ulcer	Therapy	Diffuse or not	Polypoid lesion	Other site	Pathology	Year
Fawcett [5]	36	m	Distal post	9 × 8 × 3.5 cm	None	Distal	Lobular	None	duod	Operation	1948
Peabody [6]	61	m	Distal ant	11 × 6 × 5 cm	Two	None	Lobular	None	Multiple gastric lipoma	Autopsy	1953
Deeths [8]	50	f	UML, Gre		None	Explor lap	Multiple		duod mesentery	Lap biopsy	1975
Skinner [9]	56	m	L	2.0 cm, 1.5 cm	Ulcers	Hemigastrectomy	Multiple			Opelation	1982
Ventura [10]	72	m	L	10 × 6.5 × 3 cm			Lobular	None	U, post 0.2–2 cm	Autopsy	1997
Devlies [11]	67	f	U, Gre					None	Duod	Duod biopsy	1997
Suarez-Moreno [12]	51	m							Small bowel		2010
Jeong [2]	69	f	ML, GreAntPost	16 × 16 cm	Ulcers	Subtotal	Diffuse	None		Operation	2010
Present case	54	m	UM, GreAntPost	23.0 × 14.5 cm	Ulcer	Total	Diffuse	Fundic, foveolar	None	Operation	2017

There are eight reports of gastric lipomatosis in the medical literature

*post* posterior, *ant* anterior, *U* uppper, *L* lower, *M* middle, *Gre* Greater curvature, *lap* laparoscopy, *Duod* duodenum

## Conclusions

We performed total gastrectomy for a large and ulcerated gastric lipoma in a patient with anemia. Gastric lipomatosis was confirmed by CT and EUS, which should be considered essential for diagnosis. We believe that surgery is an appropriate treatment when gastric lipomatosis is symptomatic or when there is an extensive gastric involvement or multiple gastric lipomas. This approach can also be used to confirm the diagnosis.

## Abbreviations

CT: Computed tomography
EUS: Endoscopic ultrasound
GI: Gastrointestinal
SMT: Submucosal tumor

## References

[1] Taylor AJ, Stewart ET, Dodds WJ (1990). Gastrointestinal lipomas: a radiologic and pathologic review. AJR Am J Roentgenol.

[2] Jeong IH, Maeng YH (2010). Gastric lipomatosis. J Gastric Cancer.

[3] Ferrozzi F, Tognini G, Bova D, Pavone P (2000). Lipomatous tumors of the stomach: CT findings and differential diagnosis. J Comput Assist Tomogr.

[4] Thompson WM (2005). Imaging and findings of lipomas of the gastrointestinal tract. AJR Am J Roentgenol.

[5] Fawcett NW, Bolton VL, Geever EF (1949). Multiple lipomas of the stomach and duodenum. Ann Surg.

[6] Peabody JW, Zikind J (1953). Lipomatosis of the stomach: a case report and a review of the literature. Ann Surg.

[7] Weinberg T, Feldman M (1955). Lipomas of the gastrointestinal tract. Am J Clin Pathol.

[8] Deeths TM, Madden PN, Dodds WJ (1975). Multiple lipomas of the stomach and duodenum. Am J Dig Dis.

[9] Skinner MS, Broadaway RK, Grossman P, Seckinger D (1983). Multiple gastric lipomas. Dig Dis Sci.

[10] Ventura L, Leocata P, Guadagni S, Ventura T (1997). Multiple gastric lipomas: report of an asymptomatic case found at autopsy. Pathol Int.

[11] Devlies F, Hoe LV, Leemans A, Ponette E, Paepe ID (1997). Gastroduodenal lipomatosis. Eur Radiol.

[12] Suárez Moreno RM, Hernández Ramírez DA, Madrazo Navarro M, Salazar Lozano CR (2010). Multiple intestinal lipomatosis. Case report. Cir Cir.

[13] Heiken JP, Forde KA, Golde RP (1982). Computerized tomography as a definitive method of diagnosing gastrointestinal lipomas. Radiology.

